# Recent Advances and Trends in Chemical CPP–Drug Conjugation Techniques

**DOI:** 10.3390/molecules26061591

**Published:** 2021-03-13

**Authors:** Félix Gayraud, Merlin Klußmann, Ines Neundorf

**Affiliations:** Department of Chemistry, Institute for Biochemistry, University of Cologne, Zuelpicher str. 47a, D-50674 Cologne, Germany; fgayraud@uni-koeln.de (F.G.); mklussm1@smail.uni-koeln.de (M.K.)

**Keywords:** cell-penetrating peptides, peptide–drug conjugates, bioconjugation, peptide synthesis, anticancer drugs

## Abstract

This review summarizes recent developments in conjugation techniques for the synthesis of cell-penetrating peptide (CPP)–drug conjugates targeting cancer cells. We will focus on small organic molecules as well as metal complexes that were used as cytostatic payloads. Moreover, two principle ways of coupling chemistry will be discussed direct conjugation as well as the use of bifunctional linkers. While direct conjugation of the drug to the CPP is still popular, the use of bifunctional linkers seems to gain increasing attention as it offers more advantages related to the linker chemistry. Thus, three main categories of linkers will be highlighted, forming either disulfide acid-sensitive or stimuli-sensitive bonds. All techniques will be thoroughly discussed by their pros and cons with the aim to help the reader in the choice of the optimal conjugation technique that might be used for the synthesis of a given CPP–drug conjugate

## 1. Introduction

Cancer is one of the main causes of mortality and intensive research activities on effective treatment strategies are required to tackle this severe disease. During the last years, several new anticancer drugs have been designed and developed to cure cancer. Although promising in activity, they often face various limitations such as poor targeting capacity, restricted cell penetration, or low biocompatibility, to name only few. As a consequence, efficient drug delivery has emerged as a must to achieve and is one of the main research foci in this field. Indeed, different systems have been developed for conjugating such anticancer drugs to a platform that allows for targeted delivery directly to the tumor avoiding unwanted delivery to healthy cells, thus limiting side effects. Among them, antibody–drug conjugates (ADCs) emerged as highly useful owing to their extremely selective binding affinity [1,2]. However, ADCs are often expensive to produce and have other drawbacks such as limited tissue penetration. Other strategies include the encapsulation of drugs into decorated micelles, or the use of nanoparticles as delivery modalities [3,4]. One other way that has been come more and more attractive is the design of peptide–drug conjugates (PDCs) [5,6]. They stand out by several advantages such as their good biocompatibility, cost-effective synthesis, possibility of chemical optimization, and the opportunity to assemble the system with the support of cell-penetrating peptides (CPPs).

CPPs are usually 5–30 amino acid long positively charged peptides able to overcome plasma membranes either via endocytosis or direct translocation pathways in a non-invasive manner and without disturbing the membrane integrity [5,7]. Besides, they usually have a very good tolerability and do not evoke any immunogenicity. In the last decades, CPPs were often used to generate either covalent or non-covalent drug conjugates, including a different spectrum of therapeutic molecules, such as small molecule drugs, nucleic acids (like siRNA), or proteins. Recently, liposomes or nanoparticles were additionally included creating multifunctional delivery systems [8,9,10,11,12]. While very different medical targets were accessed with the use of CPPs they have also been described to be versatile to create PDCs having anticancer drugs as payloads [13,14]. This review summarizes and discusses recent advances and trends reported in the very last years on the chemical synthesis of anticancer CPP–drug conjugates including small molecule drugs and metal complexes. Metal complexes have gained an increasing interest as anticancer therapeutics and, therefore, we will imply studies that demonstrate the efficiency of such CPP–drug conjugates. Moreover, we will concentrate on covalent bioconjugation techniques and leave out any studies on non-covalent complex formation between the CPP and drug.

Table 1 summarizes most of the CPPs mentioned within this work. For example, TAT and Penetratin are included, which are well-known and one of the first described CPPs. Penetratin is a 16 amino acid long peptide of the Antennapedia homeodomain in *Drosophila melanogaster*, which was discovered in 1994 [15]. TAT is a part of the transactivating protein of HIV-1 containing several positively charged amino acids, particularly arginine [16,17]. Inspired by the amino acid sequence of TAT, Sugiura et al. created polyarginine peptides comprising a series of several arginines ([Arg]_n_, with n = 4–16), and it was demonstrated that peptides with about eight arginine residues exhibited the best translocation efficiency [18]. However, in our group we have developed the CPP sC18 and have used it to generate various CPP–drug conjugates during the last years. sC18 is a 16 amino acid long amphipathic CPP, which is derived from the *C*-terminal region of the antimicrobial peptide CAP18 (cathelicidin). We demonstrated that sC18 exhibited comparable uptake efficiency to TAT [19] and also showed that a shortened version, namely sC18*, still exhibited valuable uptake potency [20] making it a useful drug transporter.

**Figure 1:**
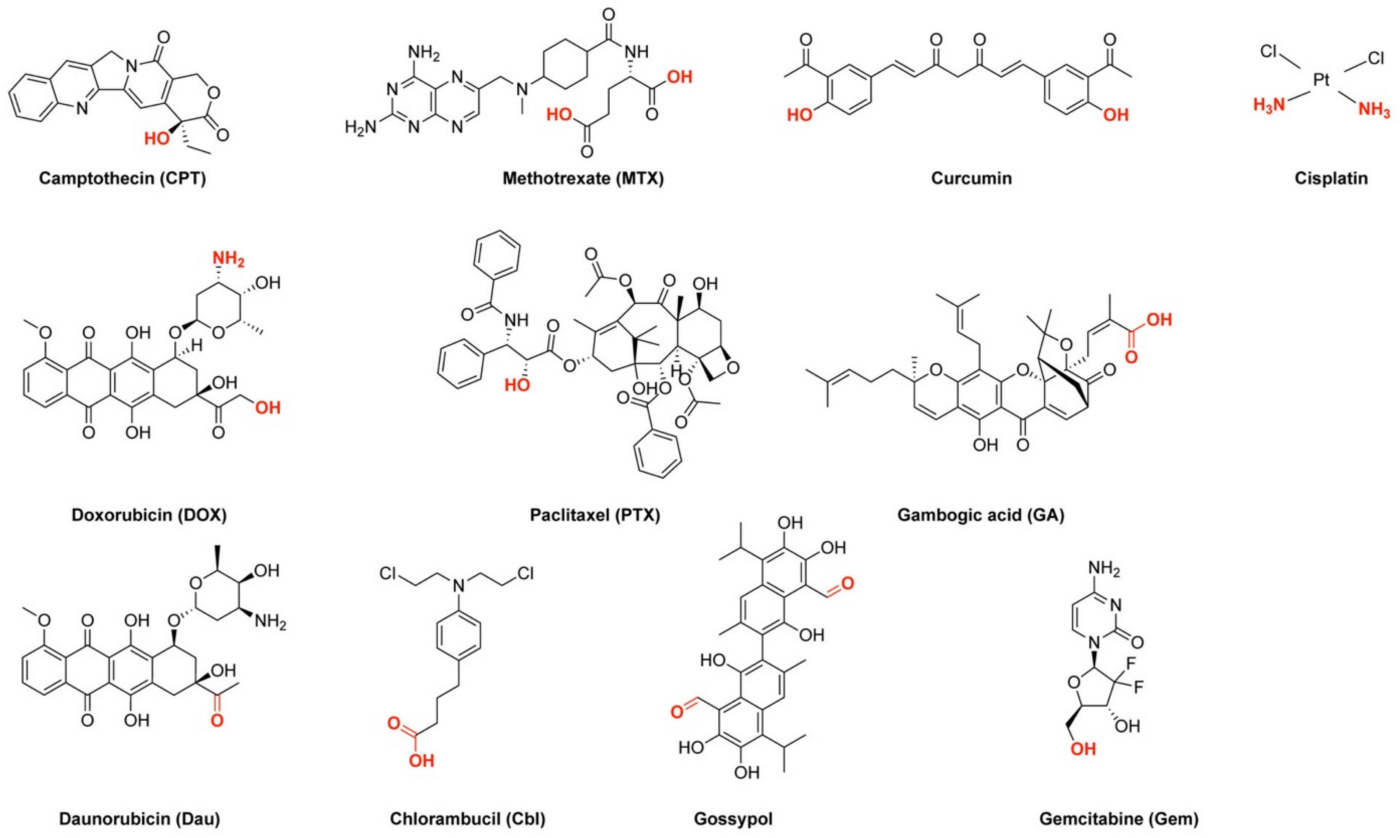
Chemical structures of bioactive molecules coupled to CPPs and discussed within this review (coupling position is highlighted in red).

Some examples of the used drugs discussed within this review are depicted in Figure 1. Many of them suffer from poor selectivity and/or water solubility which diminishes their efficiency, thus rendering the PDC strategy attractive. For example, doxorubicin (DOX), as well as daunorubicin (Dau) are well-known anticancer anthracycline drugs acting directly on topoisomerase II inhibiting DNA, RNA, or protein synthesis. However, their usage is restricted owing to their poor water solubility and undesired side effects leading to high toxicity [47]. Notably, both drugs possess an amine, which is not part of their pharmacophore, and can be used as coupling position. On the other side, camptothecin (CPT) is a natural product isolated from *Camptotheca acuminate* exhibiting anticancer potency by inhibiting topoisomerase I leading to DNA damage [48,49]. Like the previous drugs, CPT suffers from insolubility in aqueous media. A useful coupling position for CPT was observed to be the free hydroxyl group, which will then require a slightly different coupling chemistry. Methotrexate (4-amino-10-methylfolic acid, MTX) is an auspicious drug to treat resistant cancer cells. Once internalized by cancer cells, it undergoes polyglutamation by the attachment of 5–8 glutamic acid residues which makes the compound non-responsive to efflux pumps. In addition, the polyglutamylated form of MTX is able to inhibit dihydrofolate reductase and timidylate synthase, enzymes which are both involved in DNA synthesis. However, polyglutamylated MTX derivatives are highly hydrophilic compounds and, therefore, can be only poorly translocated inside cells [33]. Other drugs described herein that are utilized for generating CPP–drug conjugates will be presented within the respective paragraphs.

**Figure 2:**
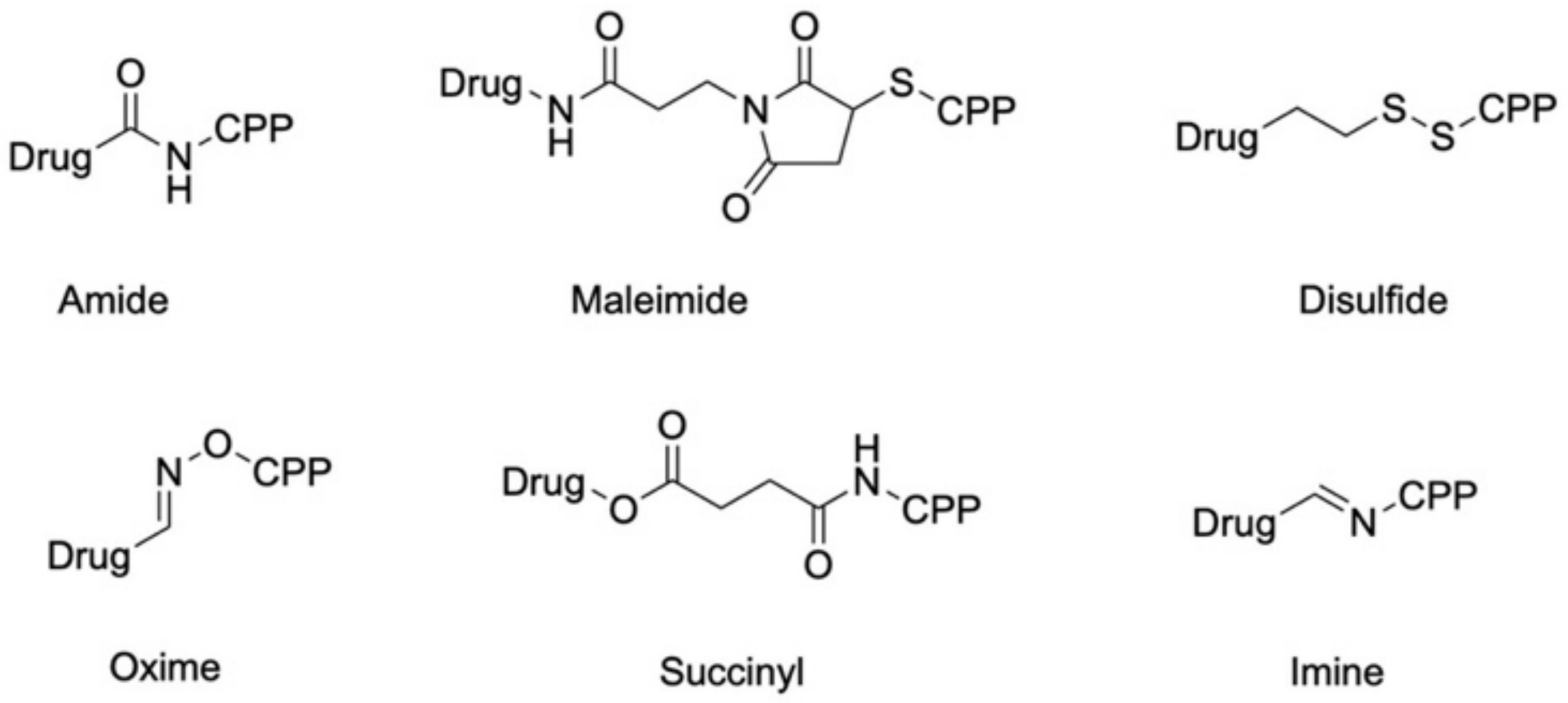
Exemplified are the different bonds formed between CPPs and drugs discussed within this work.

Over the last five years, a wide diversity of conjugation techniques have been used to conjugate drugs to CPPs. From this variety we picked out only few examples that we think are very popular and useful, e.g., amide bond chemistry and linker chemistry including maleimide, succinyl, disulfide or stimuli-reactive linkers (Figure 2). We will first describe the most basic amide bond coupling, and following, introduce more complex coupling techniques.

## 2. Amide Bond Formation and Related Chemistry

**Scheme 1:**
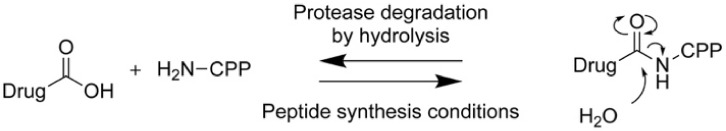
Representation of general amide bond formation and possible cleavage by proteolytic degradation.

Maybe the easiest and most efficient way to couple small molecules and metal complexes to CPPs is via amide bond formation. Similar to peptide synthesis a free amino group (often the *N*-terminus) reacts with a carboxylic acid moiety present on the drug to be conjugated (Scheme 1). The advantage of this method is that coupling steps are realized by using standard solid-phase peptide synthesis (SPPS) conditions, while the peptide is still linked to the solid support decreasing the possible formation of side products. It should be mentioned that the carboxylic acid functionality has to be activated by a suitable coupling agent (converting the hydroxyl group into a good leaving group) to achieve amide coupling. However, in many cases the drug does not bear a free carboxylic group and thus needs to be further modified for coupling to the CPP.

### 2.1. CPP–Drug Conjugates Comprising a Classical Amide Bond

The use of classical amide bond coupling to create a CPP–drug conjugate was recently presented in the work of Szabó et al. [33]. The aim was to generate a conjugate containing the drug methotrexate (MTX) with the intention to increase its bioavailability, its selective tumor delivery, and to overcome drug resistance of MTX. Therefore, they coupled pentaglutamylated MTX, which is not a substrate of the multidrug resistance protein (MRP 3) [50] and the folate transporter systems [51], to two different CPPs. The CPPs chosen were octa-arginine or a modified version of Penetratin lacking methionine at position 12 (Penetratin(desMet)), both of which offer different entry mechanisms. First, Glu_5_ was *N*-terminally coupled either directly or via a Gly-Phe-Leu-Gly (GFLG) peptide spacer to the CPPs, where in turn MTX was *N*-terminally coupled to the resulting peptide via an amide bond using HOBt-DIC as coupling agents. GFLG is a cathepsin B cleavable linker and was used since this enzyme is overexpressed in many cancer cells [52]. In vitro cellular uptake and cytostatic activity studies of the peptide–drug conjugates using drug-resistant breast cancer MDA-MB-231 and drug-sensitive breast cancer MCF-7 cells revealed that all conjugates showed internalization into both cell lines. Although the uptake of pentaglutamylated conjugates was decreased, some of the conjugates exhibited significant cytostatic activity. Interestingly, the Penetratin(desMet) conjugates were more active compared to the R_8_ conjugates. MTX-Glu_5_-Penetratin(desMet) and MTX-Glu_5_-GFLG-Penetratin(desMet) demonstrated the highest antiproliferative activity. While MTX-Glu_5_-Penetratin(desMet) only showed activity against the resistant cell line (IC_50_ = 0.1 µM), MTX-Glu_5_-GFLG-Penetratin(desMet) was active in both cell lines (IC_50_ = 0.4 µM in MDA-MB-231 and IC_50_ = 0.3 µM in MCF-7 cells). In contrast, free MTX was only toxic to drug-sensitive MCF-7 cells (IC_50_ = 0.8 µM) and showed no appreciable toxic effects to drug resistant MDA-MB-231 cells (IC_50_ > 100 µM). The efficiency of the CPP–drug conjugate was higher when the cathepsin B linker was introduced, proving again its potency for a selective drug release at the target site.

Similar to Szabó et al., the Ramakrishnan group reported on the conjugation of MTX to CPPs using classical amide bond formation [23]. As CPPs, they used different newly designed cationic helical amphipathic peptides (CHAP), which were topologically constrained to form a helical structure [53,54,55]. Such a structure is expected to improve the cell penetration efficiency. Conjugation of MTX was achieved again *N*-terminally via the two free carboxylic moieties of the drug which are not part of its pharmacophore (see Figure 1). Uptake studies revealed that the CHAP peptides were able to enter cancerous MDA-MB-231 and HeLa cells more efficiently than non-cancerous HEK-293 cells. Also, the cellular uptake mechanism seemed to be independent from energy and temperature. The CHAP–MTX conjugates showed increased cytotoxicity against the two cancer cell lines (MDA-MB-231: IC_50_ = 0.39–1.52 µM; HeLa: IC_50_ = 0.45–1.95 µM) compared to free MTX (MDA-MB-231: IC_50_ > 40 µM; HeLa = 3.99 µM). In contrast, the cytotoxic effects of the CHAP–MTX conjugates towards HEK-293 (IC_50_ between 5.20–>50 µM) cells were decreased compared to free MTX (2.24 µM) and also lower than the toxicity of the conjugates towards the two cancer cell lines, suggesting higher selectivity of the conjugates towards cancer cells.

Recently, we designed a cell-permeable mitochondria targeting chimeric peptide and conjugated it to the anticancer drug chlorambucil via classical amide bond formation [40]. Chlorambucil (Cbl) is able to alkylate DNA and was used in this approach since it has been demonstrated that targeting mtDNA, instead of nuclear DNA, results in higher mutation rates due to the lack of many DNA repair systems in cancerous cells [56,57]. The cell-permeable mitochondrial targeting chimeric peptide developed in this study comprised a mitochondrial targeting peptide derived from yeast aldehyde dehydrogenase 5 (ALD5^MTS^ (2–17)) and the CPP sC18 [40]. The sequences of the two parts were either directly coupled or via the already mentioned GFLG linker. Notably, ALD^MTS^-sC18 alone showed efficient cellular internalization and was preferably localized within the mitochondrial matrix. Moreover, only low cytotoxicity towards HeLa, MCF-7 and mouse fibroblast cells was observed. On the other hand, all peptide–drug conjugates, in which Cbl was coupled using classical amide bond chemistry, showed about a 16–20-fold increase in cytotoxicity towards HeLa cells (IC_50_-values between 1.71–2.23 µM) compared to free Cbl (IC_50_ > 35 µM), speaking in favor for an efficient delivery of the drug into the mitochondrial matrix [40].

To overcome poor water solubility of the anticancer drug gambogic acid (GA) Lyu et al. conjugated it to the CPP Tat [45]. First, an amino hexanoic acid was introduced *N*-terminally to the peptide. Then, gambogic acid was coupled via its free carboxylic moiety (see Figure 1) using unspecified amide coupling conditions. The resulting GA–TAT conjugate was characterized by highly improved water-solubility compared to GA alone. Cell assays using human bladder cancer (EJ) cells revealed significantly increased cellular uptake, cytotoxicity and apoptosis of GA–TAT. Further investigation of the underlying mechanism showed that GA–TAT increased the anticancer effect of GA alone against EJ cells by elevating the level of reactive oxygen species (ROS) resulting in ROS-mediated apoptosis. *N*-acetyl-l-cysteine, which is a well-known ROS scavenger, inhibited GA–TAT-induced ROS formation and apoptosis in EJ cells. Moreover, Western Blot analysis revealed that caspase-3 and caspase-9 were activated by GA–TAT, while the Bcl-2/Bax ratio was down-regulated. Interestingly, those effects were mostly recovered by the presence of *N*-acetyl-l-cysteine.

**Figure 3:**
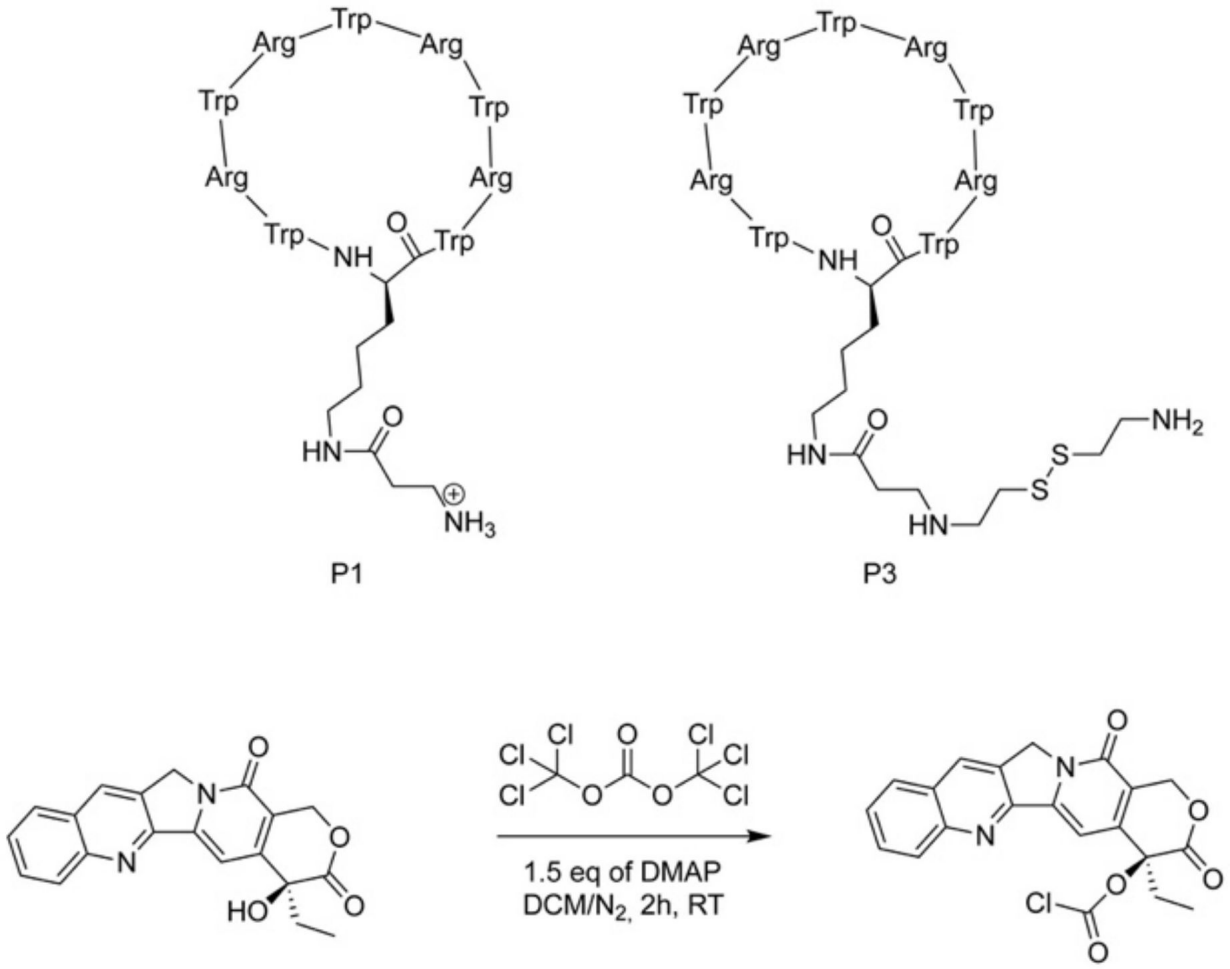
Chemical structure of the cyclic CPP P1 and P3 (after linker introduction). Chemical modification of the drug camptothecin (CPT) before its coupling, according to reference [32].

Within the next example, the presented conjugation chemistry seemed to be more sophisticated. In order to increase water solubility and drug efficiency of camptothecin (CPT), the Tiwari group coupled it to a cyclic CPP and compared two different coupling strategies; CPT was either conjugated by a classical amide bond or by a disulfide bond [32]. The used CPP (named P1) consisted of four alternating Arg and Trp residues flanked by an *N*-terminal Trp and a *C*-terminal Lys bearing a β-alanine (β-Ala) on its ε-amino group. Cyclization was performed between the *N*- and *C*-terminal (Figure 3). P1 was further modified in two steps by first using tritylthiopropionic acid in DMF/DIPEA/HBTU to introduce a thiol on the β-Ala. Subsequently, this thiol function was deprotected in order to introduce 2-[(2-pyridinyl)dithio]ethanamine via a disulfide bond as a linker to yield so called new peptide P3 (Figure 3). CPT itself offers only one hydroxyl group, which was then turned into chloroformate as a better leaving group by reacting CPT with triphosgene in DCM under N_2_ atmosphere (Figure 3). This chloroformate CPT was then either conjugated via amide bond to P1 using DMF/DIPEA (CPT1), or via disulfide bond formation to P3 using DIPEA/DMSO (CPT2). Notably, both conjugates, CPT1 and CPT2, exhibited increased water solubility compared to free CPT. Following cytotoxicity assays using breast cancer MCF-7 cells revealed that CPT2 offered a similar cytotoxicity (62% reduced cell proliferation) to free CPT (68%), while toxicity of CPT1 was significantly lower (39%). The authors explained the observed differences by the different linkage chemistry. The redox-sensitive disulfide bond is presumed to be reduced after internalization by thiols like glutathione [58], resulting in a more efficient release of the drug. On the other hand, the amide bond is more stable under physiological conditions and is degraded mainly by proteases [59], resulting in a less efficient release of the drug once internalized. This suggests that the conjugated pro-drug exhibited decreased anticancer activity than the released drug.

### 2.2. CPP–Metal Complex Conjugates

Over the last decades, metal complexes containing transition metals have received increasing attention in the treatment of many diseases, including cancer. The versatile properties of the transition metals, such as redox potential or charge variation, play a critical role in the therapeutic effects of the complexes [60]. Cisplatin (Figure 1), which was discovered by Barnett Rosenberg in the 1960s [61], and its successors carboplatin and oxaliplatin are the most prominent metal complexes used as chemotherapeutics against numerous types of cancers [61,62,63]. Inspired by cisplatin, complexes containing other transition metals such as ruthenium or cobalt were also investigated as anticancer drugs [62]. However, many potent metal complexes exhibit only poor water-solubility and poor cellular uptake [64]. Thus, conjugating the metal complexes to CPPs has been seen a promising solution to circumvent those drawbacks and to increase their bioavailability [64].

**Scheme 2:**
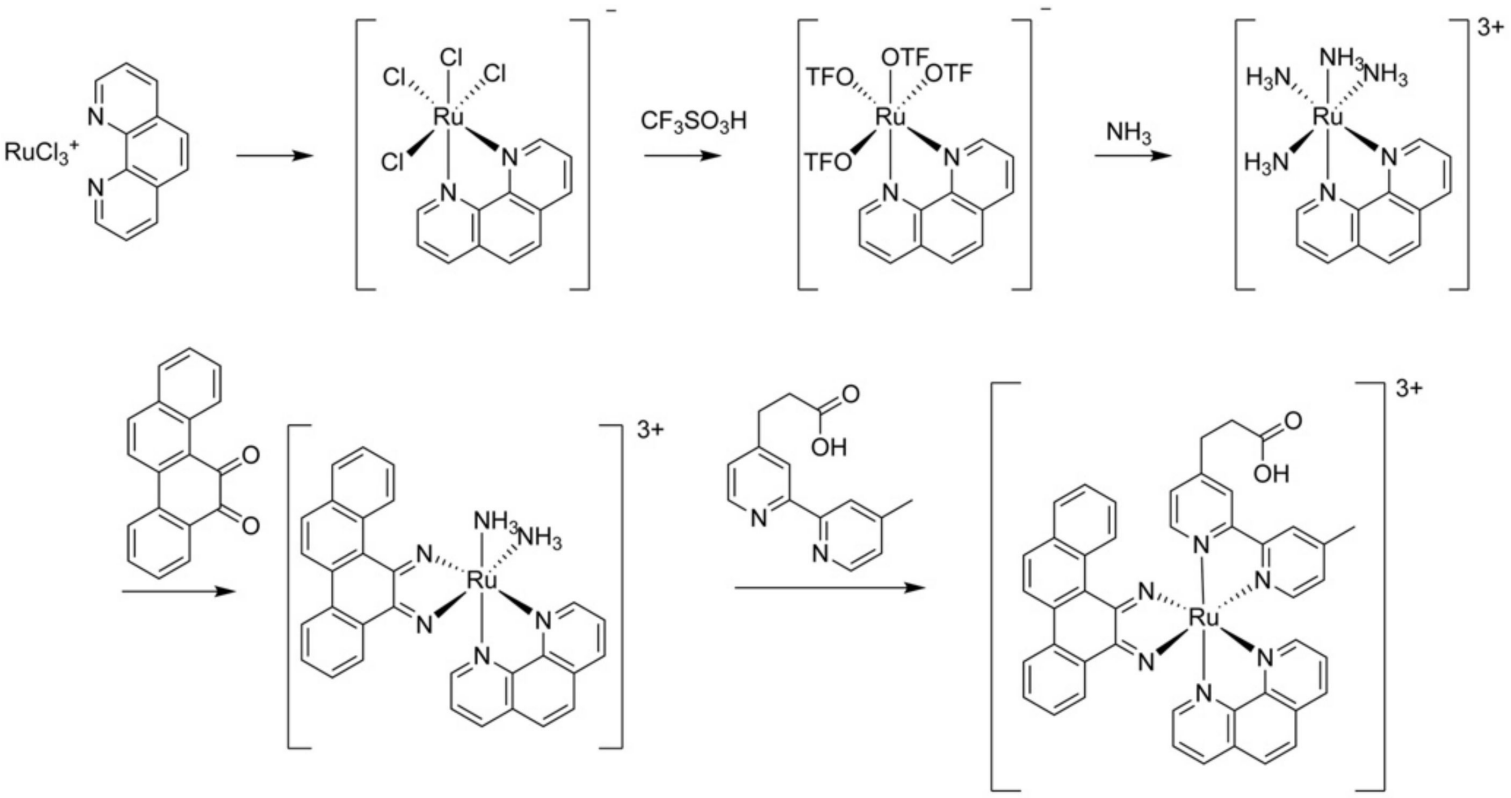
Simplified synthesis of the modified ruthenium complex, according to reference [35].

A recent example was presented by the group of Barton that developed a ruthenium metal complex targeting DNA mismatch [35]. In one of their previous studies, they attached this complex to the CPP d-octa-arginine, but the overall positive net charge provided by the CPP led to undesired non-specific uptake of the construct [65]. In order to overcome this issue, they therefore coupled the metal complex to the tetrapeptide RrRK [35], which was previously reported to accumulate in the nuclei of HeLa cells [66]. For conjugating both parts they had first to introduce a carboxylic moiety to the metal ligand. As outlined in Scheme 2, a modified bipyridine was used bearing a carboxylic moiety allowing an amide coupling to the free *N*-terminal amine of the peptide via HOBt/HBTU activation. The novel conjugate stood out due to its better water solubility compared to the free drug. Besides, presence of RrRK supported nuclear localization of the ruthenium complex, when the concentration exceeded a distinct threshold. On the other side, the threshold of RrRK was higher than that for the Ru-d-R8 conjugate indicating a general lower cell entry efficiency for the RrRK–drug conjugate. Furthermore, the influence of the payload towards delivery efficiency and localization of the peptide was demonstrated, since it was found out that RrRK complexed to Ru exhibited less delivery efficiency than RrRK complexed to thiazole orange. Thus, the metal complex seemed to negatively affect the internalization ability of the CPP.

**Scheme 3:**
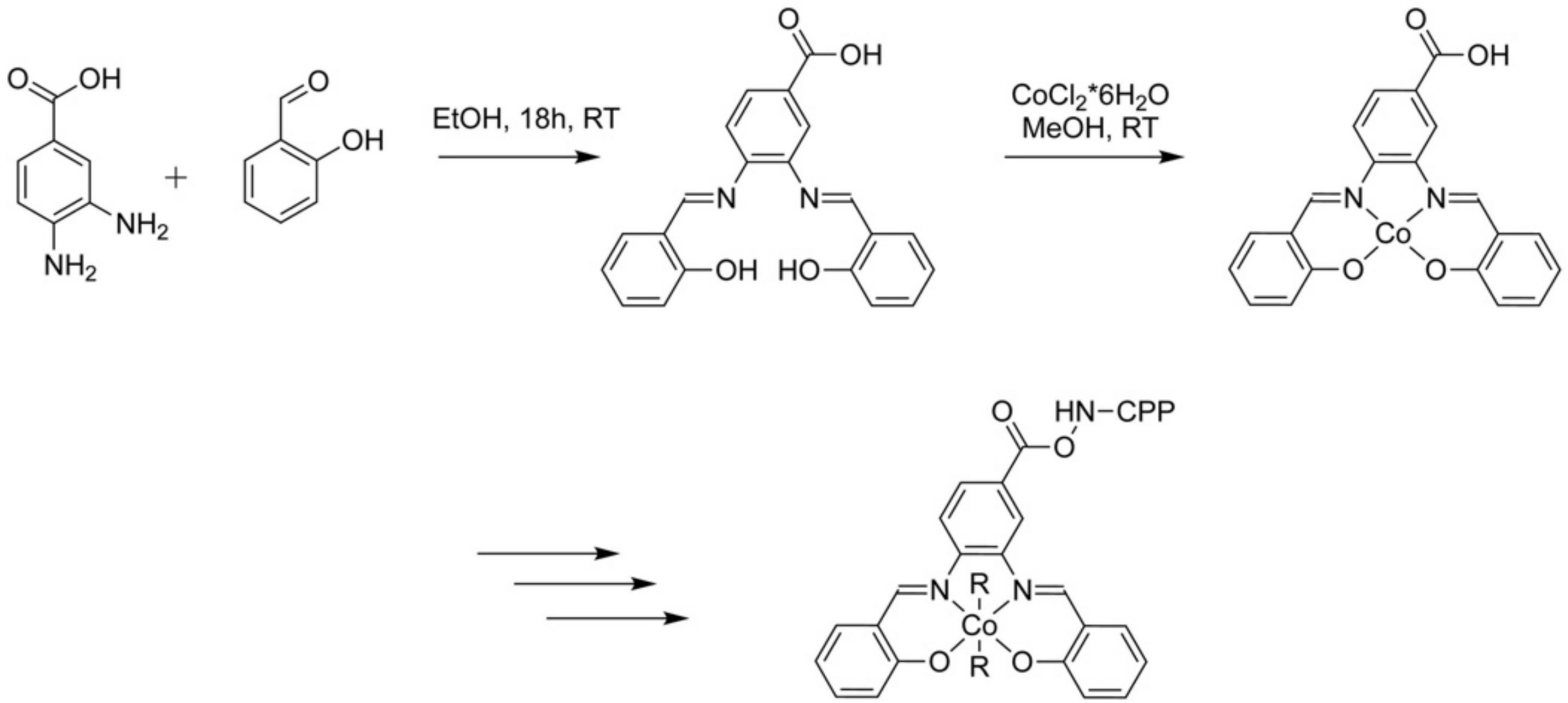
Simplified synthesis scheme of the modified cobalt complex, according to reference [37].

The Metzler-Nolte group specializes in the design and synthesis of peptide–metal complex conjugates [67,68,69]. Motivated by their long-standing experience in bioconjugation chemistry [70,71,72,73], they recently explored how to introduce cobalt complexes to peptide delivery systems, as those have yet not been extensively studied for their anticancer potential [37]. To achieve the coupling, the authors designed a Schiff base ligand by condensation of salicylaldehyde with 3,4-diamino benzoic acid (Scheme 3). This ligand comprised a carboxylic acid moiety, which was further used for coupling to the CPP via an amide bond. Afterwards, it was complexed with Co(II) and subsequently added to the *N*-donor ligand to let the complex oxidize switching from Co(II) to Co(III). As CPPs, they used three different peptides, namely FFFF, R_9_GAL and F_4_R_9_GAL, which were *N*-terminally modified with the metal complex using TBTU/HOBt activation. Following antiproliferative assays revealed that the new conjugate comprising the Co(III) complex coordinated by an l-histidine methyl ester in axial position and the CPP F_4_R_9_GAL exhibited 1.3, 1.5 and 1.4 times enhanced antitumor activity in A549, HepG2 and GM5657T cancer cells, respectively, compared to the parent Co(III) complex without the CPP.

**Scheme 4:**
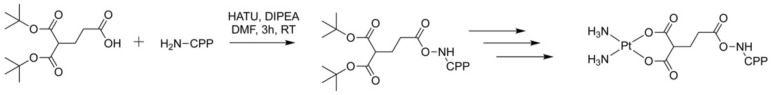
Simplified synthesis of the modified platinum complex, according to reference [22].

In another work, Parker et al. designed a PDC with synergistic properties combining the cell penetration, anti-microbial and anti-proliferative activity of the CPP buforin IIb and the well-known classical anticancer metal drug cisplatin [22]. In terms of improving stability towards proteases, the d-stereoisomeric form of buforin IIb was used. As demonstrated in Scheme 4, using HATU/DIPEA activation they functionalized the *N*-terminal amine with a malonate linker (malBuf), which offered two carboxylic moieties for further ligation to cisplatin at pH 6.6 via an amide bond. Following synthesis, the anti-proliferative effect of the metal complex–CPP conjugate (*cis*-PT[(NH_3_)_2_(malBuf_-2H_)]) was investigated in cisplatin-sensitive A2780 and cisplatin resistant A2780cisR, as well as non-cancerous NHDF cells (in comparison to cisplatin, *cis*-PT[(NH_3_)_2_(malonate)] and buforin IIb). While *cis*-PT[(NH_3_)_2_(malBuf_–2H_)] (IC_50_ = 12.2 µM) showed similar, decreased and increased cytotoxicity towards A2780 cells compared to buforin IIb (IC_50_ = 12.3 µM), cisplatin (IC_50_ = 1.3 µM), and *cis*-PT[(NH_3_)_2_(malonate)] (IC_50_ = 16.0 µM), respectively, it was significantly more toxic in A2780cisR cells compared to all other control compounds. However, the metal complex–drug conjugate exhibited also toxic effects to non-cancerous NHDF cells (IC_50_ = 7.0 µM), although this toxicity was lower compared to that of buforin IIb (IC_50_ = 5.9 µM) and cisplatin (IC_50_ = 2.4 µM).

**Figure 4:**
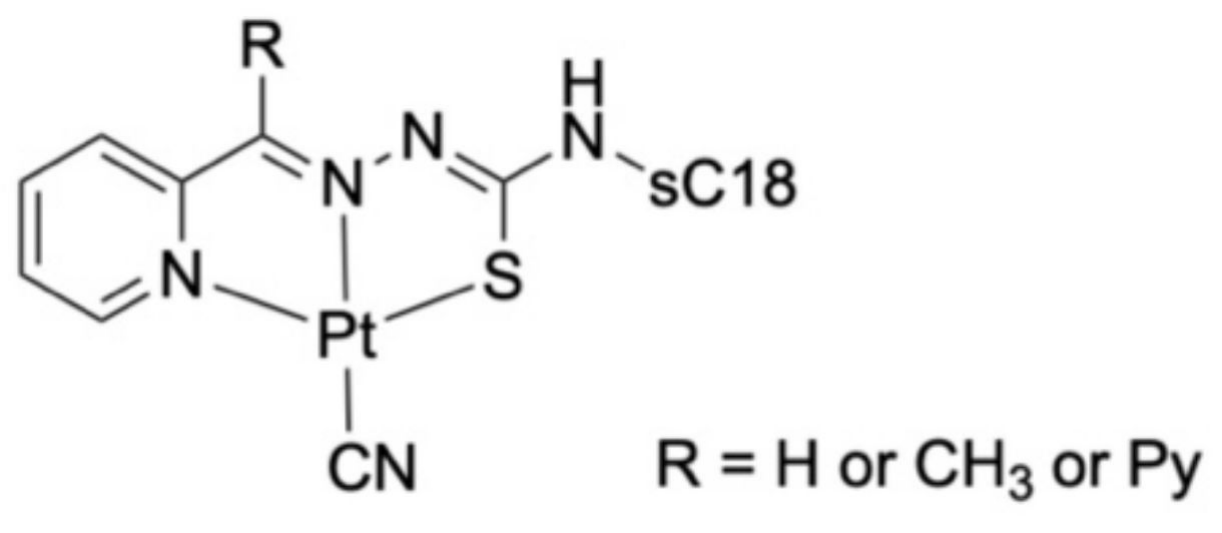
Chemical structure of the final platinum complex, according to reference [42].

Very recently, we investigated platin-containing thiosemicarbazone complexes that were introduced to the CPP sC18 (Figure 4). Also, in this case, coupling of the ligand to the CPP was achieved using SPPS via an amide bond. After cleavage from the resin and workup, the modified peptide was complexed with Pt in solution, while the final Pt(CN) derivatives were obtained through Cl^−^ to CN^−^ exchange. Since the conjugates demonstrated very good tolerability in different cell lines, they might be used in future for late-stage labelling with Pt radionuclides in nuclear medicine [42].

In conclusion, amide bond formation is a versatile option for coupling a drug to a CPP. Usually, the generated amide bond is very stable under physiological conditions and thus ensures no release of the drug while being transported to its site of action. The respective chemistry offers a wide spectrum in the choice of the adequate coupling agents including different possibilities for demanding reactions, while still being very cost-effective. Indeed, this type of bond is commonly used for the introduction of metal complexes, too, as the conditions are highly suited for this kind of compounds.

On the other hand, amide bond formation also bears several drawbacks. For instance, the amide bond being quite stable requires degradation by a protease to release the drug, since some drugs are not active as conjugates (prodrug), and this process is uncontrollable [74] (see also Scheme 1). Thus, amide bond formation between CPP and bioactive compound should be considered as an option of choice for a proof of principle study, but for effective drug delivery we will now discuss more interesting linkers and coupling chemistry.

## 3. CPP–Drug Conjugation Using Functionalized Linkers

A well-established tool to tether the CPP with the drug is the usage of functionalized linkers. When choosing the linker, however, several aspects should be considered, e.g., the stability, length, hydrophilicity, functional groups included within the structure and release mechanism [75]. This means the linker could potentially influence the efficacy of the loaded drug or the internalization efficiency of the CPP. Additionally, the linker should be stable until the drug is delivered to its site of action. In this context, this chapter highlights different types of linkers with different tumor-specific drug release strategies that have been recently used for CPP–drug conjugation.

### 3.1. Bifunctional Succinyl Linkers

**Scheme 5:**
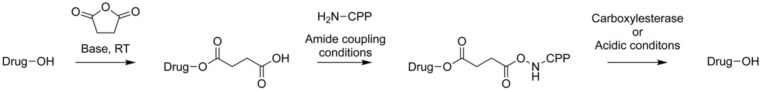
Schematic representation of succinyl linker introduction and cleavage.

The succinyl linker is a commonly used bifunctional linker based on succinic acid. Here, the drug is connected to the succinyl linker via ester bond, while amide bond coupling is used to tether the linker to the CPP (Scheme 5). The idea behind this linker is the controlled release of the drug, once internalized by the cancer cells, due to hydrolysis by carboxylesterases or under acidic conditions [75,76,77]. Following the work of Kiew et al. [78], Valizadeh and coworkers used such succinyl linker to couple gemcitabine (Gem) to variants of the CPP R_8_, in which several arginine residues were replaced by tryptophan resulting in a dramatic increased uptake into A459 cells [39]. When Gem was coupled to those peptides containing block sequences of arginine (R_5_W_3_R_4_), and some alternating Trp/Arg sequences (like [RW]_6_ and [RW]_3_) the conjugates showed indeed enhanced antitumor activity in A549 cells. In particular, cell viability was about three times lower for R5W3R3-Gem, 1.5 times for [RW]_6_-Gem and 2.9 times for [RW]_3_-Gem compared to free Gem.

Nam et al. recently explored a way of targeting breast cancer cells by using a pH-responsive PDC consisting of the CPP LH_2_ and the drugs PTX and MTX, respectively [28]. The CPP consisted of a dimer of two LH monomers, alternating Leu and His sequences, linked via a disulfide bridge between Cys at position 5 and 12. The resulting dimer was either assembled in parallel or anti-parallel hairpin structure. Histidine residues possessing a pK_a_ around 6 were supposed to get protonated specifically in tumor cell environment, resulting in a positively charged CPP with increased cellular uptake ability. PTX was introduced via a succinyl linker to the *N*-terminal lysine of the CPP, while MTX was introduced via classical amide bond coupling. The resulting LH_2_ peptides showed efficient uptake into MDA-MB-231 cells at nanomolar concentrations (10–25 nM), and particularly at acidic pH (pH 6.0–6.5). Cytotoxicity experiments towards MDA-MB-231 cells revealed that both MTX-LH_2_ and PTX-LH_2_ showed higher cytotoxicity compared to the free drugs. Interestingly, when keeping the cells at lower pH, higher toxicity values were observed, especially for PTX-LH_2_ (in the range of 50 nM). Furthermore, PTX-LH_2_ showed sustained circulation in the body and accumulated in tumors. In vivo experiments using an MDA-MB-231 mice xenograft model revealed that PTX-LH_2_ exhibited remarkable anti-tumor efficacy already at low doses (1.17 µmol/kg; injection of the conjugate three times a week). In contrast, free PTX at similar doses only showed negligible anti-tumor effects.

In conclusion, the succinyl linker is a user-friendly tool to attach a drug bearing a hydroxyl group to the amino function of a given CPP in a straightforward synthesis route. Furthermore, the length of the linker provides optimal separation of drug and peptide. Thanks to the ester bond connecting the drug with the linker, the drug, once internalized, can be easily released by carboxylesterases or under acidic conditions [39,75,77,79]. However, the stability of the ester linkage in the blood must be carefully examined to prevent unwanted drug release and off-target adverse effects [77].

### 3.2. Redox-Sensitive Disulfide Linkers

**Scheme 6:**
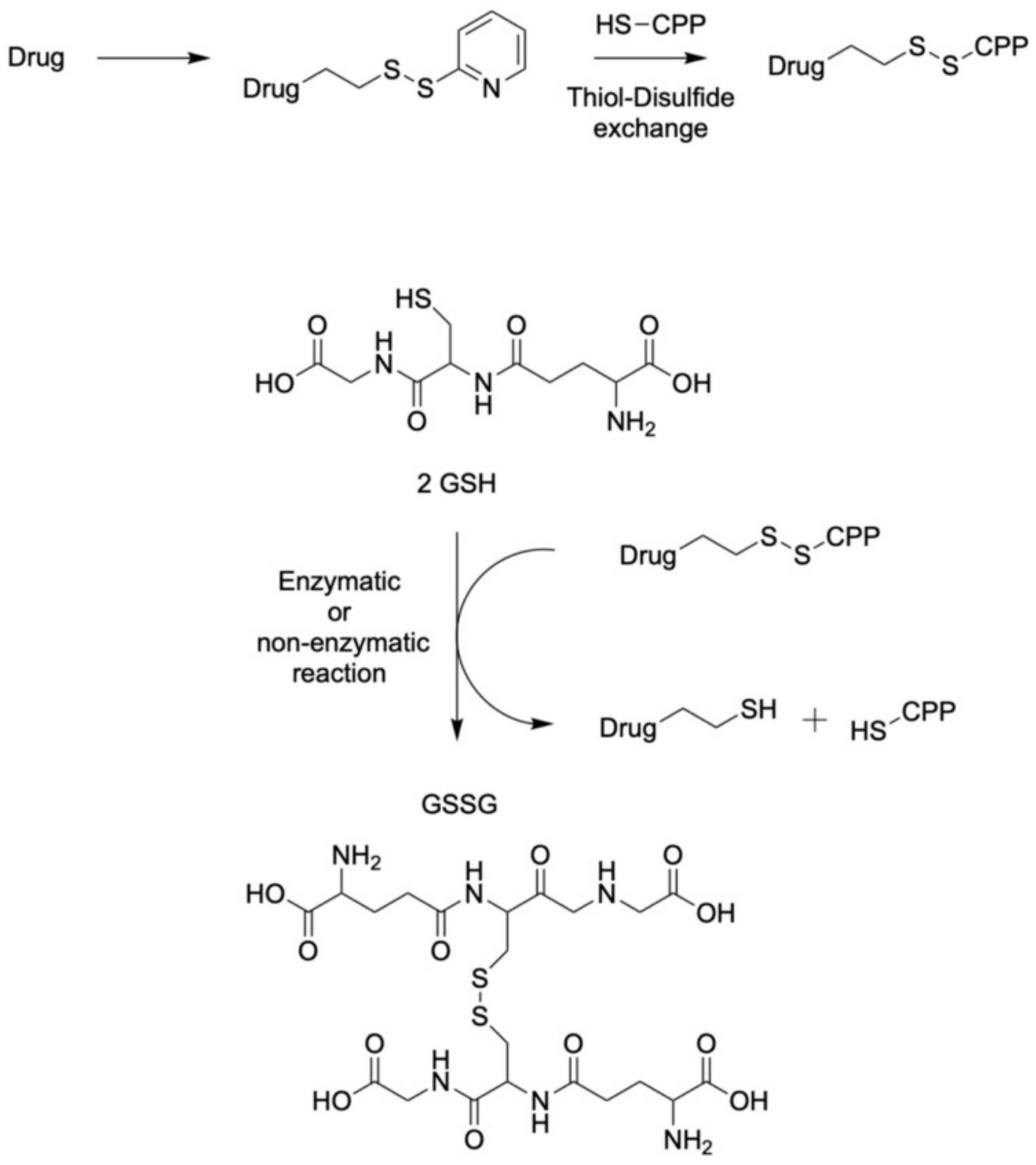
Schematic representation of disulfide linker introduction and cleavage, according to reference [81].

In order to support proper folding, proteins own disulfide bonds, which occur between two thiol moieties of cysteines. Inspired by nature, chemists often mimic this bond since it offers fast degradation within a reductive milieu (Scheme 6). Many cancer cells typically exhibit elevated levels of glutathione [58,80], and thus, this type of linker, based on 2,2′-dithiodipyridine, allows for efficient drug release by reducing the disulfide once internalized into cancer cells (Scheme 6).

For instance, Darwish et al. used this kind of bond to generate a double loaded drug–CPP conjugate. It consisted of a cyclic CPP providing improved drug delivery [24], and a double payload of curcumin and DOX, respectively, since it was already demonstrated that single drug treatment might promote drug resistance. To attach the drugs to the cyclic CPP [C(WR)_4_K_2_(β-A)] in solution, they were first modified. For this, DOX was reacted on its free amine (Figure 1) using Traut’s reagent (2-iminothiolane hydrochloride) in TEA followed by reaction with dithiodipyridine in acetic acid. Then, this DOX-SS-Pyridyl derivative was coupled to Cys of the cyclic peptide under aqueous conditions. Next, a glutaric acid spacer, which is similar to the above described succinyl spacer (Figure 2), was introduced on the free hydroxyl moiety of curcumin via a ring opening reaction in TEA/Toluene/DMAP leading to the esterified curcumin monoglutarate, which was then coupled using amide bond coupling reaction to the free amine of the cyclic peptide. For the cyclic peptide–doxorubicin and the cyclic DOX–peptide–curcumin conjugates, antiproliferative activity was investigated in human ovarian carcinoma (SKOV-3), human leukemia carcinoma (CCRF-CEM), and normal kidney cells (LLCPK). Both cyclic peptide-DOX and cyclic DOX-peptide-curcumin conjugates showed no antiproliferative activity against LLCPK cells after 72 h incubation with 5 µM of the conjugates, while they inhibited proliferation of SKOV-3 (55% and 30%, respectively) and CCRF-CEM cells (73% and 41%, respectively), indicating cell specificity of the conjugates towards cancer cell lines. In contrast, free DOX showed high cytotoxicity in all three cell lines (60–79%) under similar conditions.

In a follow-up work, Darwish et al. designed a similar CPP–DOX conjugate [25], in which DOX was coupled to the CPP by a disulfide bond and investigated in more detail the pharmacological profile of the novel PDC [58]. As CPP, they again used the cyclized peptide [C(WR)_4_K] [82,83,84]. DOX was modified prior its introduction to the CPP to circumvent sterically hindrance. As described in their previous work, Traut’s reagent was applied to thiolate DOX at its free amine. Subsequently, the amine was activated with dithiodipyridine yielding an activated DOX that was coupled to the CPP via thiolate-disulfide exchange using water as a polar, protic solvent. Following flow cytometry studies of the DOX-CPP conjugate using several different cell lines (HT-1080, HEK-293, SKOV-3 and CCRF-CEM cells) revealed that free DOX and DOX-SS-[C(WR)_4_K] showed similar uptake values, while further fluorescence microscopy studies proved high cellular uptake and mainly nuclear localization of the conjugate. Moreover, DOX-SS-[C(WR)_4_K] exhibited similar cytotoxic effects in HT-1080, HEK-293, SKOV-3 and CCRF-CEM cells compared to free DOX after 24 h of incubation. In contrast, after 72 h incubation, the antiproliferative inhibition of the conjugate was increased in HEK-293 and HT-1080 cells compared to free DOX, indicating an enhanced accumulation of DOX inside the cells based on retention of DOX by the conjugate. Furthermore, the peptide–drug conjugate provoked less cytotoxicity in mouse myoblast cells compared to free DOX. Of note was that intracellular ROS levels were low, demonstrating that there was no evidence for ROS induction after incubation with DOX-SS-[C(WR)_4_K] and Fe^2+^, while ROS production was significantly enhanced after cell treatment with free DOX and Fe^2+^.

In another study, Zhang et al. [27] also used disulfide bond coupling for generating a CPP–drug conjugate in order to profit from the oxidative release of the drug by glutathione within tumor cells. Additionally, they aimed to further increase the tumor cell specificity of their CPP LK (LKKLLKLLKKLLKL-NH_2_), which offered high potential as delivery vector due to its remarkable internalization efficiency [85,86]. Therefore, they created a LH variant where all lysine and two leucine residues were replaced by histidine. As already mentioned, the tumor surrounding is often characterized by an acidic extracellular microenvironment [87,88,89,90], which should result in protonation of the histidine residues and then, in an increased cellular uptake efficiency of the peptides [91]. However, LH was initially coupled to the anticancer drug camptothecin (CPT) via a disulfide carbonate releasable linker. The synthesis of such linker was performed according to a previously described method [92,93]. For later coupling, CPT was first modified on its 20-OH group (Figure 1) with triphosgene in DCM using an excess of DMAP as base resulting in the chloroformate form of CPT. Modified CPT was then reacted with the linker in DCM affording pyridyldithioethyl carbonate camptothecin. Finally, the CPT-linker was conjugated via the cysteine of the CPP using DMSO. Uptake studies in HeLa cells revealed that LH showed an increased internalization at pH 6.0 while the penetration activity at pH 7.4 was remarkably lower. Additionally, LH alone provoked less cytotoxicity in both pH conditions towards HeLa and MDA-MB-231 cells compared to LK. On the other side, the LH–CPT conjugate exhibited pH-dependent antitumor activity, indicating the high potential of LH as a tumor-targeting delivery vector.

Interestingly, the Wang group employed a similar strategy. As CPP, they used their previously developed pH-responsive CPP named TH (AGYLLGHINLHHLAHL(Aib)HHIL-NH_2_; Aib: 2-aminoisobutyric acid), which was shown to facilitate activated internalization into tumor cells due to protonation of its histidine residues [94]. In order to increase the protonation in acidic tumor microenvironments, the authors further modified all histidine residues of the CPP at the C-2 position by introducing different electron donating groups (ethyl, isopropyl, butyl) [46]. CPT was then coupled to the CPP at an *N*-terminal Cys via a disulfide carbonate releasable linker as it was reported in a previous publication [92]. In vitro cytotoxicity measurements in HeLa cells revealed that butyl-TH-CPT exhibited the strongest pH-response with about 50% of surviving cells when the conjugate was applied at pH 6.8, while at pH 7.4 about 65% of the cells survived.

To note is also the study of Zhang et al., in which a tumor targeting CPP–drug conjugate was designed using the so called pHLIP carrier peptide, a 36 amino acid peptide derived from the *C*-helix of bacteriorhodopsin [34]. This water-soluble peptide easily inserts into cell membranes through conformational transitions at acidic pH conditions. Indeed, the authors expected to overcome multidrug resistance of DOX thanks to this unique entry mode. Additionally, they included a glutathione (GSH)-responsive disulfide linker to support site-directed drug delivery. In fact, it was assumed that at physiological pH the CPP should only weakly interact with cell membranes, but once the pH decreases the peptide was supposed to form a transmembrane helix inserting its *C*-terminus inside the cytoplasm, where the intracellular reductive environment should reduce the linker finally leading to the release of DOX. To obtain the conjugate, they first introduced a linker to DOX through a two-step reaction. This started with an exchange reaction between 2,2′-dithiodipyridine and 3-mercaptopropionic acid in ethanol to avoid the homo-coupling of 3-mercaptopropionic acid, while an excess of 2,2′-dithiodipyridine and acetic acid was added. The resulting 2-pyridyl-2-carboxyethyl disulfide was then activated in acidic conditions using HBTU in DMF before adding DOX in its hydrochloride form and DIPEA to form the corresponding amide bond with the free amine of DOX (see Figure 1). Finally, this modified DOX was then coupled to cysteine through thiol/disulfide exchange in DMF yielding pHLIP-SS-DOX. Subsequent uptake studies using drug-sensitive MCF-7 and drug-resistant MCF-7/Adr cells revealed that the uptake of pHLIP-SS-DOX at pH 6.0 in both cell lines was about two times higher than that at pH 7.4. Also, the uptake of the conjugate at pH 6.0 was about two times higher in MCF-7 cells and about four times higher in MCF-7/Adr cells compared to free DOX. This indicated that the peptide–drug conjugate targeted tumor cells with respect to the acidic microenvironment and also harmed multidrug resistant cancer cells. Furthermore, cytotoxicity assays in MCF-7 cells revealed that pHLIP-SS-DOX at pH 6.0 with artificial elevated glutathione levels were significantly more toxic than at normal GSH levels. In contrast, no significant cytotoxic effects were detected when cells were kept at pH 7.4 with normal or artificial elevated GSH levels. In conclusion, it was demonstrated that pHLIP-SS-DOX efficiently entered tumor cells when in an acidic microenvironment, where elevated GSH levels enhanced the release of DOX from the pro-drug further increasing its overall cytotoxicity.

In terms of improving its cytotoxic profile, Lelle et al. created a DOX dimer, which was supposed to exhibit higher DNA binding affinity. For this, the DOX dimer was first linked via disulfide bond to a lysine based heterobifunctional cross-linker [95] and then coupled to the CPP octa-arginine [36]. With the synthesized peptide–drug conjugates in hand, the authors demonstrated that the dimeric DOX molecule exhibited higher binding affinity to Cy5-labeled double-stranded model DNA (7.9 × 10^6^ M^−1^) compared to single DOX (8.8 × 10^5^ M^−1^). Although the antiproliferative effect of the peptide–drug conjugate was lower in drug-sensitive MCF-7 cells (~4 times lower IC_50_) and Kelly-WT cells (~13.5 times lower IC_50_), the conjugate showed increased cytotoxicity in multi-drug resistant Kelly-ADR cells (~2.5 times higher IC_50_) compared to unmodified DOX, indicating the ability of the peptide–drug conjugate to overcome drug-resistance. The results were further confirmed by fluorescence confocal laser scanning microscopy (CLSM) studies in Kelly-WT and Kelly-ADR cells. Although the unmodified DOX was more enriched in drug-sensitive Kelly-WT cells after 72 h compared to the drug–CPP conjugate, in drug-resistant Kelly-ADR cells the signal of the conjugate was obviously stronger. Concluding the dimeric DOX conjugate demonstrated promising uptake and cytotoxic profile in drug-resistant cells.

Recently, Chen and co-workers developed a cell-penetrating and tumor-targeting peptide–PTX conjugate to increase PTX tumor specificity and membrane penetration [44]. They chose LHRH (*p*EHWSYGLRPG) as the tumor-targeting moiety, an endogenous peptide agonist targeting LHRH receptors, which is overexpressed in various tumor types [96,97], while the peptide FKKFFRKLL, which they named T2, served as the CPP. In addition, they introduced a cleavable spacer between the two peptide sequences, namely PGLAG, a linker peptide that is recognized and cleaved by cell surface enzymes such as matrix metalloproteinase-2 [98], overexpressed in many cancer cells [99]. The drug was introduced via a disulfide linker at a *C*-terminal cysteine yielding the PDC named LTP-1. Following uptake studies revealed a two-fold increased uptake of LTP-1 into LHRH overexpressing MCF-7 cells compared to free PTX, which was in-line with an observed increased cytotoxicity of LTP-1 (IC_50_: 3.8 nM; free PTX: 6.6 nM). In contrast, LTP-1 caused less cytotoxicity to non-cancerous cells (NCM460, HEK-293). Also, the cytotoxic effects of LTP-1 against paclitaxel-resistant A2780/PTX cells was about 30 times higher compared to free PTX. Further in vivo experiments in MCF-7 xenograft mice model showed increased tumor growth inhibition (83.4%) after 12 µmol/kg treatment that was significantly higher than that of PTX (65.7%) without apparent toxicities. All in all, the presented LTP-1 conjugate was characterized by its high cell uptake, good selectivity index, efficacy against drug-resistant cells and promising in vivo activity.

In conclusion, the main advantage of generating disulfide bonds between a drug and CPP are the possible cell selectivity effects based on the cleavage of the disulfide by the present intracellular glutathione. Moreover, the frequently used 2,2′-dithiodipyridine (or derivatives) is a suitable tool to ‘activate’ the drug by introducing a thiol group at the drugs site, which enables the coupling to a cysteine of the CPP via a disulfide bond.

### 3.3. Acid-Sensitive Linkers

**Figure 5:**
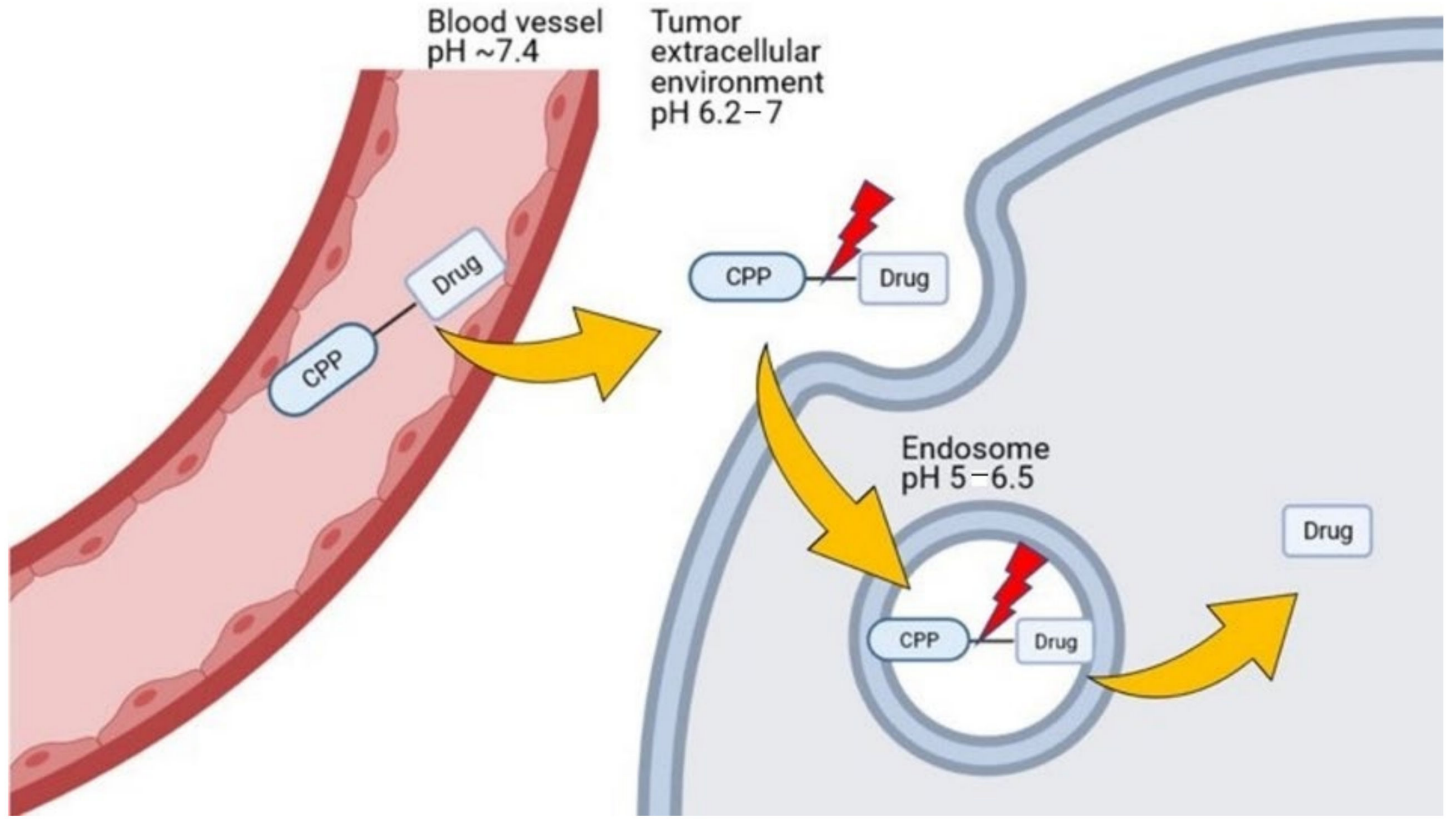
Targeting ability of an acid-sensitive linker (the lightning bolt in red represents cleavage of the linker either extracellularly or within acidic environment in endosomes/lysosomes).

Beside elevated glutathione levels, cancer cells also exhibit other characteristics that distinguish them from healthy cells. One of these characteristics are increased lactic acid production as a result of upregulated aerobic and anaerobic glycolysis. As a consequence, the extracellular tumor cell environment (pH 6.2–7.0), endosomes (pH 5.0–6.5) and lysosomes (pH 4.5–5) become more acidic than healthy tissues and blood (pH ~7.4) [76,100,101]. Based on these properties, some acid-sensitive linkers have been designed to facilitate tumor-specific drug release of CPP–drug conjugates (Figure 5), which will be highlighted in the following paragraphs.

**Scheme 7:**
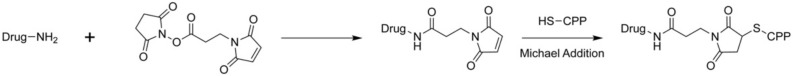
Representation of maleimide linker introduction.

Acid-sensitive bifunctional linkers bearing a maleimide are currently one of the most famous and frequently used strategies for drug conjugation. A fact that is reflected in the huge number of publications that are found in this area. Usually, the linker part consists of a maleimide group, introduced via amide bond chemistry directly onto the drug or via a succinyl spacer, which is then connected to the CPP via a Michael addition. Such reaction thus requires the presence of a free thiol, usually delivered by a cysteine in the CPP sequence (Scheme 7). Interestingly, the growing interest in the use of the maleimide linker relies on its acid sensitivity, which results in specific cleavage within the tumor environment. In fact, the thioether linkage is stable at physiological pH but undergoes rapid hydrolysis and subsequent drug release when the pH drops to more acidic values. Of course, this has raised several concerns related to the use of maleimide linkages in ADC research, where maleimide is still one of the most popular choices [102].

However, in a recent study, Yu et al. designed a new CPP named KRP [103] and loaded it with DOX through a maleimide linker [29]. The aqueous solubility of DOX was increased due to the high hydrophilicity of KRP, and increased selectivity was expected thanks to the enhanced permeability and retention (EPR) effect. The EPR effect is based on physiological and biochemical differences between many tumors and normal tissues. To supply tumors with nutrients and oxygen, new blood vessels are often formed (angiogenesis). However, these new blood vessels are usually abnormal in their vascular structure. Compared to normal capillaries, the endothelial cells of these newly formed blood vessels exhibit wide fenestrations and are therefore more permeable for macromolecules. Additionally, tumors generally show deficiencies in the lymphatic system, which prevents effective lymphatic drainage, resulting in a longer retention of macromolecules in tumors [104,105,106]. The synthesis of the KRP–DOX conjugate proceeded according to a previous method [107]. Shortly, DOX was first reacted with 3-(maleimido)propionic acid *N-*succinimidyl ester (BMPS) and triethylamine as base in DMF resulting in DOX-Mal which was then coupled to KRP via an *N*-terminal cysteine. Fluorescence microscopy studies of the resulting conjugate partly deciphered the uptake mechanism in cancerous osteosarcoma MG63 cells. It was suggested that after endocytic uptake the conjugate escaped the endolysosomes and accumulated in the nuclei. The subsequent preliminary in vivo studies delivered promising results, since from 30 min to 48 h after intravenous administration of KRP-DOX into xenograft osteosarcoma mice, no free DOX was detected within circulating blood. Simple diffusion was effectively replaced by the EPR effect resulting in significant tumor growth inhibition, and after 28 days the tumor volume of KRP–DOX treated mice were about five times lower compared to control mice [103].

Using the same method Zhang et al. reported about coupling of DOX to a so-called activatable CPP (ACPP) [21]. To decrease undesired DOX delivery to healthy cells, they introduced a masking group, 2,3-dimethylmaleic anhydride (DMA), to all ε-amino groups of present lysine residues. At physiological pH, DMA is negatively charged, allowing the formation of non-covalent bounds with arginine in the sequence, compacting and bending the PDC. On the other side, when reaching the tumor microenvironment, DMA degradation readily occurs uncaging the positive charges of lysine, thus triggering selective cell penetration of the PDC in tumor cells. DOX-ACPP-DMA internalization was followed in HeLa and COS7 cells using CLSM and flow cytometry, and the authors recognized a significant increase in fluorescence when mimicking the tumor microenvironment (by a pH drop from 7.4 to 6.8). In vitro cytotoxicity studies using the same two cell lines revealed a pH-dependent toxicity, in which about 40% of tumor cells were effectively targeted by the novel conjugate while at physiological pH no toxicity was observed even when higher concentrations of the PDC were applied. In vivo anti-tumor evaluation using H22 xenografted mice revealed that DOX-ACPP-DMA exhibited a significant inhibitory effect on tumor growth. The observed tumor volumes were 40 times smaller compared to the control, and almost no noticeable DOX side effects were monitored.

Similarly, Qi et al. developed a masking strategy to trigger the selectivity of a nona-arginine CPP when in the vicinity of cancerous cells [38]. Based on matrix metalloproteinase 2 and 9 (MMP-2/9), frequently overexpressed proteases in tumor environment [108], they constructed a masked CPP–DOX conjugate. DOX was modified with BMPS and introduced via a present *C*-terminal cysteine. A cleavage motif, the peptide PLA*LAG, was introduced *N*-terminally to nona-arginine, and additionally, a negatively charged sequence was coupled onto it resulting in a β-hairpin masked PDC (mCPP–DOX). The cleavage peptide was then expected to be specifically attacked by MMP-2/9 setting free the nona-arginine–DOX conjugate only at the tumor site, and further triggering its cellular uptake and subsequent targeted drug release. Peptide cleavage was assessed in vitro verifying the efficient MMP-2/9 degradation with the following Michaelis–Menten kinetic parameters: K_M_ = 6.61 µg/mL; V_m_ = 38.61 µg/(mL × h). Additional cytotoxicity studies confirmed the sensitivity of mCPP–DOX to MMP-2/9-rich tumor cells, with the IC_50_ value for the masked conjugate being two times lower in MMP-2/9-rich HT-1080 cells than in MMPs-depleted MCF-7 cells. Following in vivo studies using a HT-1080 xenograft mice model showed efficient tumor growth inhibition (about 100%) at a dose of 15 mg/kg DOX equivalents similar to free DOX at a dose of 5 mg/kg (each 3 times treated). However, the survival rate of the mCPP–DOX treated mice after 20 days (>65%) were significantly higher compared to free DOX (~35%), indicating fewer side effects of the conjugate. Finally, mCPP–DOX exhibited a more controllable internalization into MMP-rich tumor cells, and remarkable in vivo toxicity decrease towards normal tissues compared to free DOX and unmasked CPP–DOX.

Aside from DOX, other recent examples in which drugs were coupled using the maleimide linker include e.g., PTX, which was conjugated recently by Wang et al. to TAT or to the newly described CPP LMWP, respectively [109,110]. For this, PTX was first modified at its free hydroxyl group (Figure 1) using succination [30]. TAT possesses three free amino groups which are required for its activity [111], therefore PTX was coupled in this case by maleimide linkage to a cysteine introduced beforehand. Compared to free PTX both PDCs created showed significantly increased cellular uptake in the two investigated cell lines A549 and A549T, respectively. Moreover, PTX–TAT and PTX–LMWP exhibited increased cell toxicity compared to free PTX in both cell lines studied. Further assays revealed that both conjugates increased cell apoptosis (especially LMWP–PTX) and decreased mitochondrial membrane potential. Additionally, cell cycle analysis suggested that LMWP–PTX followed a different mechanism influencing mitosis in drug-resistant cancer cells compared to free PTX and TAT–PTX. Finally, compared to PTX alone, in A549 tumor-bearing mice tumor growth inhibition was more effective for the PDC with an increased inhibition rate about 63% for PTX–LMWP and PTX–TAT indicating a promising in vivo antitumor efficacy of the PDC conjugates.

**Scheme 8:**
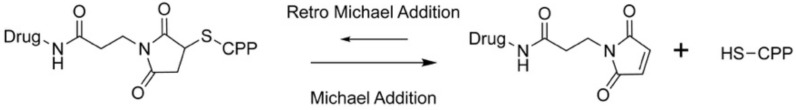
Degradation of maleimide bond that can occur in physiological conditions.

Thanks to its acid sensitivity bifunctional maleimide linkers have been extensively used for tumor targeting in many applications. Beside the requirement of a cysteine within the CPP sequence, the drug needs the presence of a free amine for modification, independent from its pharmacophore, a condition which is not always available. Apart from this, its introduction on the drug requires basic conditions and an excess of the drug, a premise which might be difficult to meet. Finally, what is often not mentioned in the cited studies is the fact that maleimide linkers can undergo a reverse Michael addition at physiological conditions, resulting in a degraded linker and unwanted release of the drug at undesired sites (Scheme 8). Thus, “stronger” maleimide linkers were developed by several groups and recently reviewed elsewhere [102]. In conclusion, the maleimide linker might be interesting owing to its targeting potency, but more work is needed to increase its targeting capability and cell selectivity.

**Scheme 9:**
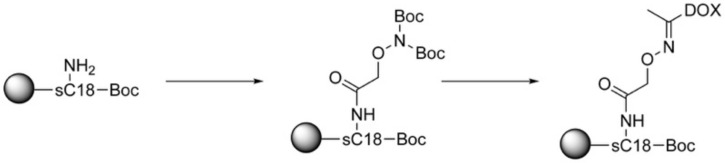
Simplified introduction of the oxime ligation, according to reference [41].

Another acid-sensitive linker strategy relies on oxime bearing bifunctional linkers (Figure 2). This kind of bond is supposedly stable at physiological pH (pH 7.4) but is cleaved at pH lower than 5.5–5.0, as encountered in tumor cells [112,113,114]. In recent work, we also used this conjugation strategy for the synthesis and biological evaluation of CPP–drug conjugates. Since CPPs lack cell selectivity, we created in a first approach a tumor targeting delivery system. Therefore, we considered the fact that integrin α_v_β_3_ receptors are highly expressed on several cancerous cells and can be effectively targeted by the tripeptide sequence RGD [115,116,117,118]. Starting from this, a peptide–drug conjugate was designed bearing a RGD targeting ligand, a CPP, namely sC18, and the classical anticancer drug daunorubicin [41]. The RGD targeting unit was based on a cyclized diketopiperazine scaffold and ligated to the CPP via copper (I) catalyzed alkyne-azide click chemistry. Beforehand, sC18 was loaded with Dau. For this, the side chain of a lysine was used for the introduction of a bis-Boc aminooxyacetic acid using 3 eq excess of DIC and Oxyma (see Scheme 9). After cleavage from resin the peptide was dissolved in ammonium acetate buffer at pH 5 before introducing a 30% excess of Dau for oxime ligation.

In vitro affinity studies of the conjugates on isolated tumor-associated integrin receptors α_v_β_3_ and α_v_β_5_ revealed that the conjugates exhibited a considerably higher affinity to α_v_β_3_ receptors compared to α_v_β_5_. Different uptake studies of c[DKP-RGD]-PEG_4_-sC18(Lys_8_-CF) (10 µM) into U87 cells, which are reported to overexpress integrin α_v_β_3_ receptors [119], in the presence of the integrin ligand c[DKP-RGD] (competing with the conjugate for the integrin receptors), poly(_L_-lysine) (masking the negatively charges of the outer leaflet of the cell membranes) or methyl-β-cyclodextrin (agent for cholesterol depletion) indicated that the conjugates were supposedly recognized by the α_v_β_3_ receptor, but then preferably taken up by the cells via CPP-mediated cellular internalization. Investigation of the cytotoxic effects of all Dau-loaded conjugates on U87, HT-29 (lacking expression of α_v_β_3_, expression of α_v_β_5_) [119] and MCF-7 (decreased expression of α_v_β_3_) cells by incubating different concentrations of the conjugates for 72 h showed high toxicity in all cell lines (IC_50_ value in low micromolar range). The results of the cytotoxicity assay supported the conclusion from the uptake studies, since there was no remarkable difference in cytotoxicity between the tested cell lines. In order to investigate whether rapid binding of the integrin targeting unit to the surface receptor would enhance the selectivity of the conjugates, uptake and cytotoxicity studies with shorter contact times (15 min of incubation before wash-out) were performed. The c[DKP-RGD]-PEG_4_-sC18(dau = Aoa − Lys_8_) conjugate showed significantly increased internalization and cytotoxicity for U87 cells compared to HT-29 and MCF-7 cells. In summary, the internalization of the conjugates was mainly mediated by the CPP, while the selectivity towards α_v_β_3_ integrin receptor expressing cells was supported by the targeting moiety.

In another study, we investigated a cyclic variant of a shorter version of sC18 (sC18*) with the idea to increase the translocation efficiency of sC18* and thus create a more efficient shuttle for anticancer drugs. The cycle was based on a 3*S*,6*R*-2,5-diketopiperazine (DKP3) scaffold, and we also coupled Dau to the side chain of lysine 4 of sC18* via a bifunctional oxime linker [43]. In order to test the biological activity of the conjugate, cellular uptake studies and cell viability assays were performed. For reasons of comparison, a linear sC18*-dau conjugate was also synthesized. CLSM studies in HeLa cells revealed that after 30 min incubation with 5 µM of the conjugates, both the linear and cyclic conjugate showed a cytosolic and nucleic distribution, while the uptake of the cyclic one seemed to be more efficient. This was also confirmed by flow cytometry experiments, where the cyclic conjugate exhibited a three-fold increased uptake into HeLa cells after 30 min incubation with 10 µM of the conjugates. This trend was in line with the cytotoxicity studies, since the cyclic conjugate (IC_50_ = 9.3 µM) exhibited a two-fold decreased IC_50_ value compared to the linear conjugate (IC_50_ = 20.1 µM).

Oxime linkers have not been used as frequently as maleimide linkers in recent years. The reason for this might be that their introduction is somehow more laborious compared to the maleimide linkage. In fact, in order to introduce an oxime linkage linker, aminooxyacetic acid is a proven tool, reacting with a primary amine of the CPP. Subsequently, the primary amino group of aminooxyacetic acid reacts with a ketone or aldehyde of the drug to form an oxime. For this, however, the drug requires an aldehyde or ketone, which is not always the case.

In addition to bifunctional maleimide or oxime linkers, imine-containing linkers have also gained some attention. Here, an aldehyde or a ketone of the drug reacts with a primary amine (*N*-terminus or ε-NH_2_ group of a lysine residue) to form such a bond (Figure 2). Imine linkers are supposed to be stable under physiological pH but undergo hydrolysis when the pH decreases (pH 4.5–6.5) [120]. For example, Maity et al. [31] coupled gossypol, an attractive ROS-inducing drug, via an imine linker to a CPP recently developed by Matsushita and co-workers [121]. The CPP chosen (RLYMRYYSPTTRRYG) was especially designed to target MCF-7 breast cancer cells, which are also the target of gossypol. Conjugation was achieved with an excess of gossypol. The reason was that it possesses a symmetrical structure with two aldehyde moieties (Figure 1), and the excess was required to avoid a second coupling. After peptide dissolution in 6 M Gn·HCl buffer at pH 5, gossypol was coupled *N*-terminally to an Ala via imine formation using 5 mol excess in methanol under gentle heating (45 °C). With the synthesized gossypol–CPP conjugate in hand, the authors tested the cytotoxic effect to MCF-7 (derived from breast cancer), HeLa (derived from cervical cancer) and Wi-38 cells (normal human fibroblasts). The conjugate showed selective cytotoxicity on MCF-7 cells since after 48 h incubation with 100 µM of the conjugate, only 50% of the MCF-7 cells were alive while HeLa and Wi-38 cells exhibited about 80% viability. Furthermore, the time dependent gossypol release of the conjugate was investigated in 6 m Gn HCl buffer (pH 7) at 37 °C via time dependent HPLC analysis. Both the CPP and gossypol were cleaved from the linker with a half-life of about 10 h. With this result, they assumed that if the conjugate has similar stability in the cytoplasm, the 10 h half-life would be sufficient for the conjugate to accumulate within MCF-7 cells before the drug is released.

Although rather rarely used in recent years, one advantage of imine linkers is that they are traceless. This means that after cleavage of the drug, there is no structural change in the original substance and consequently no restriction of the drug efficacy is to be expected. However, similar to the oxime linkers, the drug requires an aldehyde or ketone to be coupled, which is not always present in the structure.

The different types of drug release from the linkers mentioned so far all rely on the presence of internal stimuli (such as increased glutathione levels or increased acidity in the tumor cell environment). However, there are also more complex linkers that release the drug in response to external stimuli such as temperature, magnetic field, ultrasound or light [76]. As an example, Zang et al. designed a new light-responsive self-immolative linker, namely PC4AP, for DOX conjugation to the H3 mutant H3-V35C CPP [26]. This linker was a derivate of deoxyribose with the C4 and C hydroxyl groups *O*-nitrobenzyl protected. Under light exposure (365 nm), those nitrobenzyl groups were cleaved triggering addition/elimination reactions resulting in the release of DOX and cyclization of the peptide without any trace of the linker. Evaluation of the stability of the PC4AP linker demonstrated that after 24 h incubation at physiological pH (7.5) and acidic pH (5.2) the linker remained stable. On the other side, irradiation of the conjugate at 365 nm removed the photolabile *O*-nitrobenzyl groups after 3 min and subsequent addition of 20 equivalents of benzyl amine resulted in the complete release of DOX after a further 5 min. Finally, cell growth inhibition of HeLa cells was investigated when incubating the cells with 5 µM solutions of H3-PC4AP-DOX. Notably, subsequent photoirradiation caused 20% cell growth inhibition, while no inhibition was measured without irradiation. This again confirmed the stability of the conjugate within the cells in the absence of irradiation that would minimize off-target effects as a result of unwanted drug release.

This study impressively demonstrated the potential of linkers responding to external stimuli and that they are exceptionally well suited for site-directed drug release. However, unlike other well-studied linkers, such novel systems are difficult to design, and their in vitro and in vivo properties must first be critically investigated. That is probably why until now, not much work was found in this field for the synthesis of CPP–drug conjugates.

## 4. Conclusions and Future Perspectives

Undoubtedly, coupling a drug to a CPP offers several advantages for the drug like improved water solubility, increased stability and higher permeability, all prerequisites for an efficient cancer therapeutic. Many techniques for coupling the drug to a CPP have been developed over the past 3–5 years, however, we observed two major conjugation strategies that were frequently followed: direct coupling and the involvement of functionalized linkers. Direct coupling is mainly achieved via amide bond chemistry, the main benefit of which is from its straightforward synthesis route. In our opinion, this method is highly valuable for performing proof of principle studies, since the products are relatively easy to obtain. Regarding the conjugation of metal complexes, amide bond formation is the method of choice since the introduction of other functionalities to the ligands are often relatively demanding and might influence the metal complex activity.

Besides, we found that three linker categories were mainly described during the last years for the generation of CPP–drug conjugates, including disulfide redox-sensitive, acid-sensitive and stimuli-responsive linkers. Disulfide linkers are very popular as they offer cell selectivity towards cancerous cells, and also their chemistry is well-established. Different acid-sensitive linkers have been presented like imine, oxime and maleimide linkages. This acidic sensitivity is a useful way to achieve cell selectivity by specifically targeting the acidic tumor microenvironment. Finally, stimuli-sensitive linkers were introduced as more sophisticated systems, offering the chance to tailor the conjugate according to specific needs. Although it is possible to gain remarkable results with those linkers, the outcome very much depends on the conditions used and it is probably questionable how they perform in in vivo systems and if they would deliver reproducible data.

To conclude, different points must be considered in the choice of a CPP–drug ligation strategy:

1. What functional group within the drug structure is available for coupling to the CPP?
  It is important to know which groups define the pharmacophore, what positions are useful for modifications, and which reaction conditions are selectable.
2. How does the coupled drug influence the CPP?
  It should be carefully considered which cargo moieties are allowed to be introduced that do not disturb the activity of the CPP.
3. Can the tumor/tumor environment be targeted by the chosen linkage strategy?
  The microenvironment of the tumor tissue is distinguishable to that from healthy cells, and thus, linker systems should be introduced between CPP and drug that enable the generation of a highly active and cell selective anticancer therapeutic.

In any case, the design of the perfect CPP–drug conjugate is highly challenging and still might not be accessible in near future, however, it is allowed to shoot for the stars, and to aim for the moon.

## Figures and Tables

**Table 1:** Overview of the cell-penetrating peptides (CPPs) discussed within this review. Underlined sequences describe cyclization; small letters stand for the d-conformation of the respective amino acids; Aib: 2-aminoisobutyric acid; Kβ-A: beta-alanine is coupled to the ε-NH_2_ group of lysine via amide bond.

Name	Sequence	Citation
**ACPP**	RRRRRRRRGGGPKKKKKK	[21]
**Buforin IIb**	RAGLQFPVGRLLRRLLRRLLR-NH_2_	[22]
**CHAP-1** **CHAP-2** **CHAP-3**	KR-Aib-IRLFTK-Aib-LK KR-Aib-IRLFTK-Aib-FK KR-Aib-FRLFTK-Aib-FK	[23]
**[C(WR)_4_K_2_(β-A)]**	RCKβ-ARWRWKWRW	[24]
**[C(WR)_4_K]**	RCRWRWKβ-AWRW	[25]
**H3-V35C**	ARTKQTARKSTGGKAPRKQLATKAARKSAPATGGCKK	[26]
**LH**	LHHLLHHLHHLLHH	[27]
**LH_2_ (monomer)**	Acetyl-LHHLCHLLHHLCHLAG-NH_2_	[28]
**KRP**	CSSKEKKKGKKRKKKREREGQKEGGRRKEKRKEKKRKEGGREGRKEGRKSADHPS	[29]
**LMWP**	VSRRRRRRGGRRRR	[30]
**MCF-7 targeting CPP**	RLYMRYYSPTTRRYG	[31]
**P1**	Kβ-AWRWRWRWRW	[32]
**Penetratin(desMet)**	RQIKIWFQNRRKWKK	[33]
**pHLIP**	AEQNPIYWARYADWLFTTPLLLLDLALLVDADEGTCG	[34]
**Nucleus targeting CPP**	RrRK	[35]
**l** **-R_8_**	RRRRRRRR	[33,36]
**d** **-R_8_**	rrrrrrrr	[35]
**R_9_GAL** **FFFF** **FFFFR_9_GAL**	RRRRRRRRRGAL FFFF FFFRRRRRRRRRGAL	[37]
**LGA-** **d** **-R_9_C**	LGA-rrrrrrrrrC	[38]
**[RW]_6_** **[RW]_3_** **R_5_W_3_R_3_**	RWRWRWRWRWRW RWRWRW RRRRRWWWRRR	[39]
**sC18**	GLRKRLRKFRNKIKEK-NH_2_	[40,41,42]
sC18*	GLRKRLRKFRNK	[43]
**T2**	FKKFFRKLL	[44]
**TAT**	CGGGYGRKKRRQRRR	[30,45]
**TH**	AGYLLGHINLHHLAHL-Aib-HHIL-NH_2_	[46]

## Abbreviations

A2780	human ovarian carcinoma cells
ADC	antibody–drug conjugate
CCRF-CEM	human leukemia carcinoma cells
CLSM	confocal laser scanning microscopy
COS7	primate fibroblast-like kidney cells
DCM	dichloromethane
DIC	N,N’-diisopropylcarbodiimide
DIPEA	N,N’-diisopropylethylamine
DMA	2,3-dimethylmaleic anhydride
DMAP	4-dimethylaminopyridine
DMF	dimethylformamide
DMSO	dimethyl sulfoxide
EJ	human bladder cancer cells
GSH	glutathione
HBTU	hexafluorophosphate benzotriazole tetramethyl uronium
HEK-293	human embryonic kidney cells
HeLa	human cervical cancer cells
HOBt	hydroxybenzotriazole
HT-1080	human fibrosarcoma cells
HT-29	human colon cancer cells
IC50	half maximal inhibitory concentration
LLCPK	porcine kidney cells
MCF-7	human breast cancer cells
MDA-MB-231	epithelial human breast cancer
MG63	human osteosarcoma
MMP-2/9	metalloproteinase 2 and 9
MRP	multidrug resistance protein
mtDNA	mitochondrial DNA
NCM460	normal human colon mucosal epithelial cells
NHDF	normal human dermal fibroblast cells
PDC	peptide–drug conjugate
ROS	reactive oxygen species
SKOV-3	human ovarian cancer cells
TEA	triethylamine
U87	human primary glioblastoma
Wi-38	normal human fibroblast-like lung cells
